# Complete Genome Sequence of Hydrogen Peroxide-Producing Limosilactobacillus fermentum DM072 Isolated from the Human Oral Cavity

**DOI:** 10.1128/mra.00897-22

**Published:** 2022-11-02

**Authors:** Do-Young Park, Jiyoung Hwang, Yunji Kim, Young-Youn Kim, Hye-Sung Kim, Inseong Hwang

**Affiliations:** a DOCSmedi Co., Ltd., Goyang-si, South Korea; b Apple Tree Institute of Biomedical Science, Apple Tree Medical Foundation, Goyang-si, South Korea; University of Maryland School of Medicine

## Abstract

The complete genome of hydrogen peroxide (H_2_O_2_)-producing Limosilactobacillus fermentum strain DM072, isolated from the oral cavity of healthy volunteers in South Korea, was sequenced by long-read sequencing and was subsequently corroborated by short-read sequencing. The genome comprises one circular chromosome and one plasmid and lacks antimicrobial resistance genes.

## ANNOUNCEMENT

To isolate strains beneficial to oral health, we obtained human tongue coatings from healthy Korean donors between September 2021 and December 2021. H_2_O_2_-producing colonies were identified by cultivation on 3,3′,5,5′-tetramethylbenzidine-de Man, Rogosa, and Sharpe (TMB-MRS) agar for 24 h at 37°C (1, 2). A colony suppressing Streptococcus mutans, a cariogenic oral pathogen, was selected by coculture on MRS agar plates (3), and the species was identified by 16S rRNA sequencing by Macrogen, Inc. (South Korea) in February 2022.

For whole-genome sequencing, the isolate was anaerobically and statically cultivated for 24 h at 37°C in MRS broth. Genomic DNA (gDNA) was extracted using the Maxwell RSC system (Promega, USA). For PacBio sequencing, gDNA (3 μg) was sheared to 7 to 12 kb using the Megaruptor 3 (Diagenode, USA) followed by a cleanup using the AMPure PB beads (PacBio, USA). The Sequel system equipped with SMRT cells 1 M v3 tray (PacBio) was used to sequence the libraries constructed using the SMRTbell express template prep kit 2.0 (PacBio), generating 145,301 subreads with an *N*_50_ length of 10,272 bp and SMRT cells 1 M v3 tray with an average length of 8,422 bp. The reads were assembled using the Microbial Assembly protocol in SMRT Link v10.1.0.119588 (PacBio) (4), resulting in an *oriC*-rotated circular chromosome and a plasmid (Table 1). Adaptive focused acoustic technology (Covaris, USA) was used to shear gDNA (100 ng) for Illumina sequencing. The sequencing library was constructed using the TruSeq Nano DNA high-throughput library prep kit 2.0 (Illumina, USA) and analyzed on the HiSeq X Ten system (Illumina). A total of 9,501,060 bp with a Phred score (5, 6) over 30 in ≥90% of the 2 × 151-bp paired-end reads were subjected to error correction and adapter trimming with Trimmomatic v0.38 (7). The paired-end data were then mapped against the PacBio assembly using BWA-MEM v0.7.17 and polished three times using Pilon v1.21 (8) with a mindepth of 0.01.

**TABLE 1:** Summary of assembly and annotation statistics for L. fermentum DM072

Genetic element	Length (bp)	GC (%)	No. of coding sequences	No. of rRNAs	No. of tRNAs	Sequencing depth (no. of reads)	GenBank accession no.
Chromosome	2,060,072	51.7	2,035	15	58	506.9	CP102714
Plasmid pLFDM072	44,665	40.2	53			1,043.7	CP102715
Total	2,104,737	51.4	2,088	15	58	518.3	

OrthoANI (9) analysis identified Limosilactobacillus fermentum IMDO130101 (GenBank accession no. NZ_LT906621) as the closest strain, with 99.47% sequence identity. Genome annotation using PGAP v6.2 (10) reported 2,088 protein-coding genes, 15 rRNA genes, and 58 tRNA genes. Antimicrobial resistance genes were absent in DM072 according to ResFinder v4.1 (11) and CARD v3.2.3 (12). All tools were run with default parameters unless otherwise specified.

The biospecimens used in this study were provided by the Biobank of Apple Tree Dental Hospital after approval from the public institutional review board (IRB; approval no. P01-202111-31-002; http://public.irb.or.kr).

### Data availability.

The 16S rRNA gene sequence, genome sequence, and the raw sequencing reads for DM072 were deposited in GenBank (accession nos. OP579180, CP102714, and CP102715), BioProject (accession no. PRJNA852394), BioSample (accession no. SAMN29328592), and SRA (accession nos. SRX15900883 and SRX15900884).
